# Supplementary material for: Extranodal Extension Predicts Poor Survival Outcomes among Patients with Bladder Cancer
Source: Cancers (Basel). 2021 Aug 15;13(16):4108. doi: 10.3390/cancers13164108 (PMC8391350; doi:10.3390/cancers13164108)
Supplement: Supplementary file 1 [file cancers-13-04108-s001.zip › cancers-1345458-supplementary.pdf]

Supplementary Materials: Extranodal Extension Predicts Poor Survival Outcomes among Patients with Bladder Cancer

Yi-An Liao, Chun-Ju Chiang, Wen-Chung Lee, Bo-Zhi Zhuang, Chung-Hsin Chen and Yeong-Shiau Pu

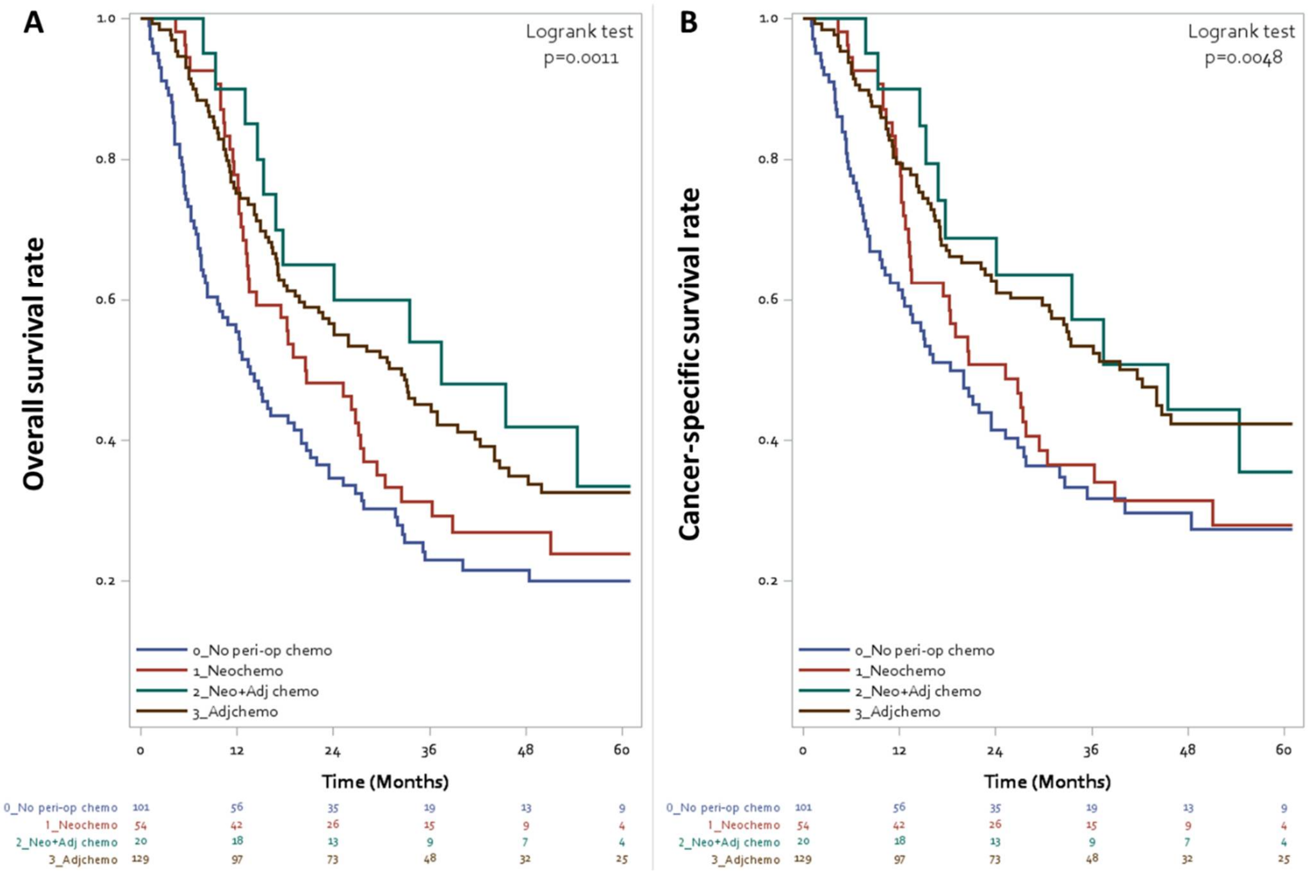

Figure S1. Overall survival and cancer-specific survival curves stratified by peri-operative chemotherapy (4 treatment categories) in bladder cancer patients with lymph node metastases treated with radical cystectomy.

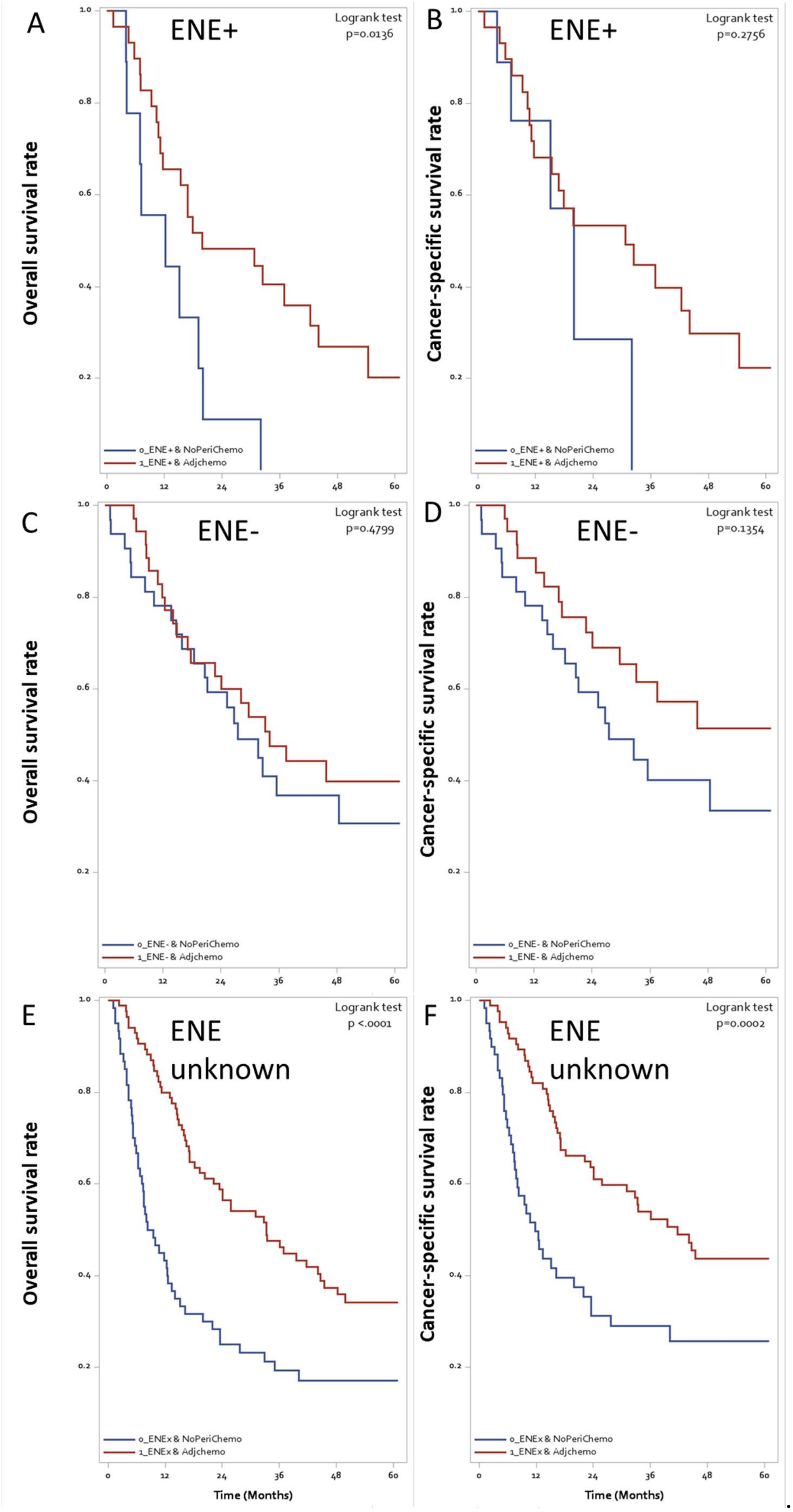

Figure S2. The impact of adjuvant chemotherapy on overall survival and cancer-specific survival stratified by the status of extranodal extension in bladder cancer patients with lymph node involvement treated with radical cystectomy.

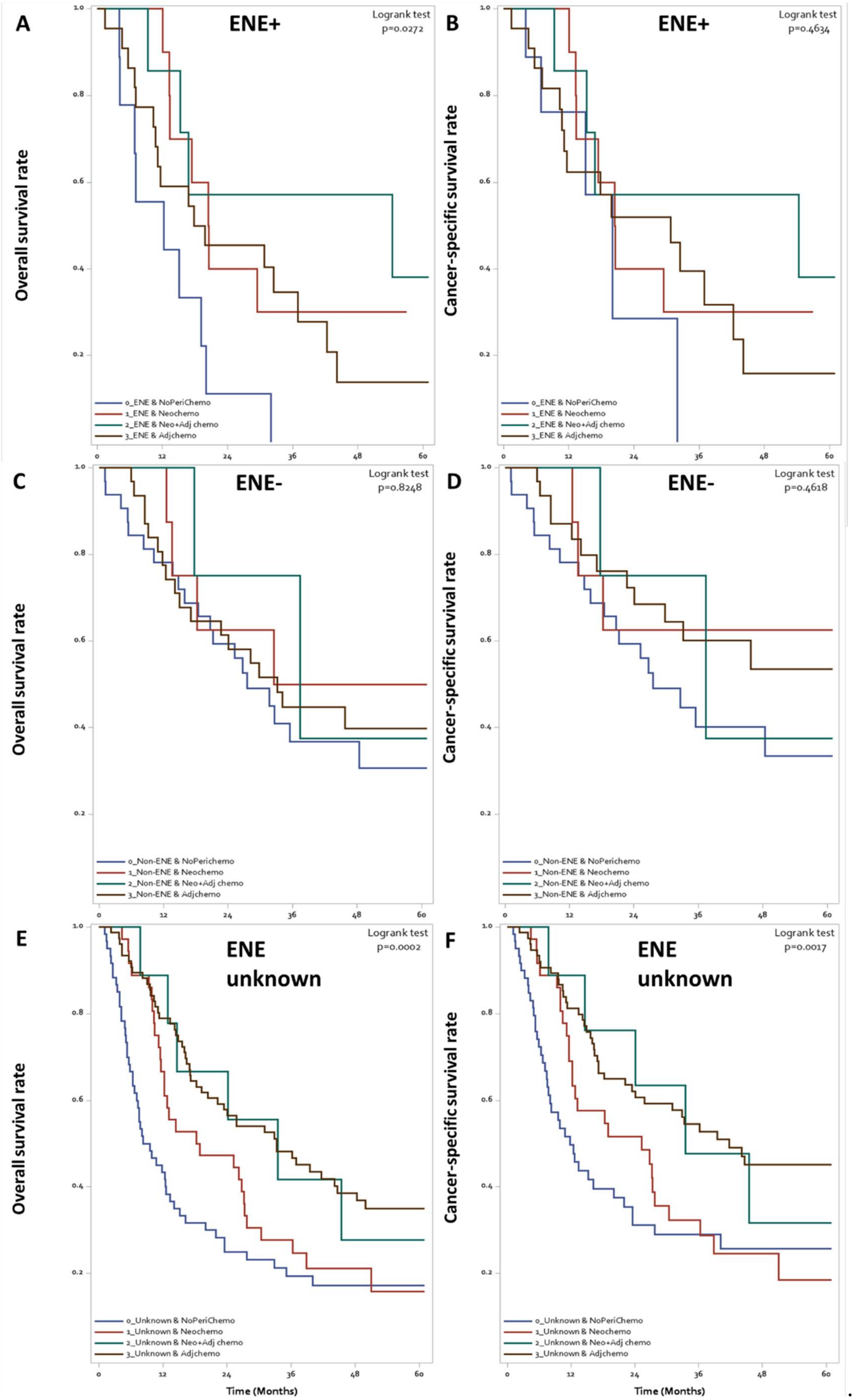

Figure S3. The impact of peri-operative chemotherapy on overall survival and cancer-specific survival stratified by the status of extranodal extension in bladder cancer patients with lymph node involvement treated with radical cystectomy.

Table S1. The Cox proportional hazard model of overall and cancer-specific survival with 4 categories of perioperative chemotherapy in bladder cancer patients with lymph node involvement who received radical cystectomy.

Variables.	Case number	All-cause death	Cancer-specific death	Univariable analysis						Multivariable analysis					
				Overall survival			Cancer specific survival			Overall survival			Cancer specific survival		
				HR	95%CI	P value	HR	95%CI	P value	HR	95%CI	P value	HR	95%CI	P value
Age (years)															
25–64	136	79	69	1	(ref)		1	(ref)		1	(ref)		1	(ref)	
65–74	111	84	68	1.55	1.14–2.11	0.005	1.43	1.02–2.0	0.04	1.48	1.08–2.03	0.02	1.36	0.96–1.91	0.08
>= 75	57	49	38	2.4	1.66–3.42	<0.001	2.11	1.4–3.13	<0.001	2.15	1.46–3.16	<0.001	1.82	1.18–2.78	0.006
Gender															
Male	214	150	120	1	(ref)		1	(ref)		-	-	-	-	-	-
Female	90	62	55	0.98	0.72–1.31	0.89	1.08	0.78–1.48	0.62	-	-	-	-	-	-
Tumor size (cm)															
< 4	88	54	42	1	(ref)		1	(ref)		1	(ref)		1	(ref)	
>= 4	155	115	100	1.55	1.13–2.16	0.008	1.73	1.22–2.51	0.003	1.55	1.12–2.18	<0.001	1.70	1.18–2.49	0.005
Unknown	61	43	33	1.3	0.86–1.93	0.21	1.28	0.81–2.01	0.29	1.21	0.76–1.9	0.42	1.03	0.60–1.74	0.91
Tumor grade															
Low	3	2	2	1.13	0.19–3.53	0.87	1.34	0.22–4.21	0.68	-	-	-	-	-	-
High	301	210	173	1	(ref)		1	(ref)		-	-	-	-	-	-
Pathological T stage															
T0	9	4	4	1	(ref)		1	(ref)		1	(ref)		1	(ref)	
Tis/a/1	6	4	3	1.55	0.37–6.57	0.53	1.17	0.23–5.32	0.83	2.53	0.53–12.1	0.23	1.91	0.34–9.86	0.44
T2	47	24	19	1.13	0.44–3.84	0.82	0.90	0.34–3.09	0.84	1.49	0.50–5.5	0.50	1.32	0.43–5.06	0.65
T3	156	109	94	1.96	0.82–6.38	0.19	1.69	0.71–5.52	0.30	2.66	0.95–9.63	0.09	2.46	0.85–9.05	0.13
T4	86	71	55	2.71	1.12–8.89	0.05	2.10	0.86–6.93	0.15	3.56	1.26–12.9	0.03	2.94	1.01–10.9	0.07
Surgical margin															
Free	247	164	136	1	(ref)		1	(ref)					-	-	-
Not free	50	42	34	1.66	1.16–2.30	0.004	1.61	1.09–2.32	0.01				-	-	-
Unknown	7	6	5	2.03	0.8–4.19	0.09	2.05	0.72–4.5	0.12				-	-	-
Lymph node density															
< 20%	148	88	67	1	(ref)		1	(ref)		1	(ref)		1	(ref)	
>= 20%	142	114	98	1.83	1.38–2.43	<0.001	2.06	1.51–2.82	<0.001	1.50	1.12–2.02	0.01	1.74	1.25–2.43	0.001
Unknown	14	10	10	1.63	0.79–2.98	0.14	2.14	1.04–3.97	0.02	2.11	0.96–4.20	0.046	2.85	1.27–5.79	0.01

Extranodal extension															
No	75	44	36	1	(ref)		1	(ref)		1	(ref)		1	(ref)	
Yes	48	37	31	1.59	1.02–2.46	0.038	1.62	1.0–2.63	0.048	1.74	1.09–2.78	0.02	1.70	1.01–2.84	0.045
Unknown	181	131	108	1.5	1.07–2.13	0.02	1.51	1.05–2.23	0.03	1.80*	1.27–2.61	0.001	1.81*	1.23–2.73	0.003
Multiple primaries															
No	248	178	151	1	(ref)		1	(ref)		-	-	-	-	-	-
Yes	56	34	24	0.76	0.52–1.08	0.14	0.63	0.4–0.95	0.04	-	-	-	-	-	-
Smoking history															
Never	188	130	109	1	(ref)		1	(ref)		-	-	-	-	-	-
Current	67	45	39	0.97	0.68–1.34	0.84	1	0.68–1.43	1	-	-	-	-	-	-
Ever	46	36	26	1.47	1–2.10	0.04	1.26	0.81–1.91	0.28	-	-	-	-	-	-
Unknown	3	1	1	0.34	0.02–1.52	0.28	0.41	0.02–1.84	0.38	-	-	-	-	-	-
Body mass index															
< 18	54	34	26	0.72	0.48–1.06	0.11	0.68	0.43–1.05	0.09	-	-	-	-	-	-
18–23.9	121	91	74	1	(ref)		1	(ref)		-	-	-	-	-	-
24–26.9	77	54	47	0.87	0.62–1.22	0.43	0.93	0.64–1.34	0.71	-	-	-	-	-	-
>= 27	52	33	28	0.71	0.47–1.04	0.09	0.74	0.47–1.13	0.17	-	-	-	-	-	-
Peri-operative chemotherapy															
No	101	78	63	1	(ref)		1	(ref)		1	(ref)		1	(ref)	
NAC alone	54	40	36	0.73	0.49–1.06	0.1	0.81	0.23–1.21	0.32	0.65	0.42–0.98	0.04	0.75	0.47–1.17	0.21
AC alone	129	82	65	0.56	0.41–0.77	<0.001	0.55	0.39–0.78	<0.001	0.54	0.38–0.76	<0.001	0.51	0.35–0.74	<0.001
NAC plus AC	20	12	11	0.47	0.23–0.83	0.016	0.54	0.27–0.98	0.049	0.53	0.26–1.00	0.06	0.65	0.30–1.27	0.23

CI = confidence interval; HR = hazard ratio; ref = reference; NAC = neoadjuvant chemotherapy; AC = adjuvant chemotherapy. * In the consideration of unknown status of extranodal extension (ENE) as reference, positive ENE was not associated with poorer overall (HR = 0.97, 95% CI 0.66–1.41) and cancer-specific survival (HR = 0.94, 95% CI. 0.61–1.41).
